# Utilisation des contraceptifs par les femmes rurales mariées ou en concubinage au Burkina Faso: une analyse qualitative de l’utilisation d’un bon gratuit

**DOI:** 10.11604/pamj.2020.37.72.23786

**Published:** 2020-09-18

**Authors:** Richard Bakyono, Ludovic Deo Gracias Tapsoba, Aurélia Lépine, Abdramane Berthé, Patrick G Ilboudo, Cheick Omar Diallo, Nicolas Méda, Ben D’Exelle

**Affiliations:** 1 Observatoire National de la Santé de la Population, Ouagadougou, Burkina Faso,; 2 Centre MURAZ, Bobo-Dioulasso, Burkina Faso,; 3 University College London, London, United Kingdom,; 4 Université de Dédougou, Dédougou, Burkina Faso,; 5 Independent Researcher, Ouagadougou, Burkina Faso,; 6 Institut de Recherche en Sciences de la Santé, Ouagadougou, Burkina Faso,; 7 Université Joseph Ki-Zerbo, Ouagadougou, Burkina Faso,; 8 University of East Anglia, Norwich, United Kingdom

**Keywords:** Planification familiale, bons, Burkina Faso, Family planning, vouchers, Burkina Faso

## Abstract

**Introduction:**

les faibles niveaux d’utilisation des contraceptifs en Afrique de l’Ouest sont responsables de taux de fécondité élevés, ce qui limite le développement économique. Le coût des contraceptifs modernes étant une contrainte importante, le gouvernement du Burkina Faso a décidé de rendre la planification familiale gratuite. Avant cette nouvelle politique, nous avons fourni aux femmes rurales un bon qui leur donnait un accès gratuit aux contraceptifs modernes. L’analyse des déterminants de l’utilisation du bon fournit des informations pertinentes pour le potentiel de la nouvelle politique gouvernementale.

**Méthodes:**

six mois après la distribution des bons aux femmes dans 30 villages de la province du Houet, nous avons réalisé des focus groups avec des participantes à l’étude et des entretiens individuels approfondis avec des prestataires de soins dans les centres de santé partenaires.

**Résultats:**

les avantages de la planification familiale, la gratuité des contraceptifs, l’approbation de leurs maris et l’obligation morale étaient les raisons de l’utilisation du bon par les femmes. Le désir de tomber enceinte, l’opposition de leurs maris (aux contraceptifs), la réticence des femmes, la méconnaissance des contraceptifs par les femmes et les facteurs liés à l’intervention ont été évoqués comme les raisons de la non utilisation des bons.

**Conclusion:**

la promotion des contraceptifs modernes auprès des couples mariés ou en concubinage exige une approche holistique, combinant à la fois le libre accès aux contraceptifs modernes, la conception de politiques efficaces pour impliquer les hommes dans la planification familiale, et la réduction des préférences de fertilité des couples.

## Abstract (en)

**Introduction:**

low levels of contraceptive use in Western Africa are responsible for high fertility rates, which limits economic development. The cost of modern contraceptives is a significant constraint, then the government of Burkina Faso has implemented free family planning. Given this new policy, we provided rural women with a healthcare voucher giving free access to modern contraceptives. We conducted an analysis of the determinants of good free voucher use in order to implement adequate government policy.

**Methods:**

six months after the distribution of vouchers to women living in 30 villages in the Houet Province, we conducted a focus-group study based on individual in-depth health care provider interviews in partner healthcare centers.

**Results:**

the benefits of family planning, free contraceptive use, husband’s approval and moral obligation were factors facilitating voucher use. The desire to become pregnant, husband’s opposition, women’s reluctance, women’s lack of knowledge of contraceptives and factors associated with the intervention were the leading reasons for not using the vouchers.

**Conclusion:**

the promotion of modern contraceptive use among married women or concubines requires a holistic approach combining free access to modern contraceptives, effective policies involving men in family planning and the reduction of fertility preferences among the couples.

## Introduction

La population mondiale était d’environ 7,5 milliards de personnes en 2017 et 17% de celle-ci était composée d’africains [1]. L’Afrique fournira plus de la moitié de la croissance anticipée de la population globale sur la période allant de maintenant à 2050 [1]. Cette situation pourrait s’expliquer à la fois par le maintien des taux élevés de fécondité et l’accroissement des espérances de vie. En effet, en 2016, l’indice synthétique de fécondité (ISF) en Afrique sub-saharienne était en moyenne de 5 enfants par femme contre 2,5 enfants par femme à l’échelle mondiale [2]. En outre, les dix pays ayant les ISF les plus élevés au monde se situent en Afrique avec en tête le Niger (7,6 enfants par femme). Le Burkina Faso occupait la onzième place avec un ISF de 5,7 [2]. Une fécondité élevée induit un accroissement plus rapide de la population et un déséquilibre de la pyramide des âges avec une part importante de la population des moins de 15 ans. Pourtant, il existe une forte corrélation positive entre la transition démographique et le développement économique [3, 4]. Au Burkina Faso, les moins de 15 ans représentaient 46,6% de la population totale en 2006 [5].

Malgré les nombreuses initiatives tendant à améliorer le recours à la planification familiale (PF) telles que la subvention et la distribution à base communautaire des produits contraceptifs, la prévalence contraceptive moderne en 2016 était de 13% en Afrique occidentale contre 46% et 59% respectivement en Afrique du Nord et en Afrique du Sud. Au Burkina Faso, cette prévalence était de 20% pour la même année [2]. Le coût des contraceptifs modernes est une des causes des besoins non satisfaits des femmes en matière de PF [6]. Ainsi, le gouvernement burkinabè a décidé de la gratuité de la PF dont l’opérationnalisation a commencé en juin 2019 dans les régions du Centre-Ouest et des Cascades. Cette nouvelle politique devrait s’étendre à l’échelle nationale et contribuerait à augmenter la prévalence contraceptive dans le pays. Toutefois, l’utilisation des méthodes contraceptives modernes par les africaines en union dépend de plusieurs autres facteurs majeurs [6-10].

Les études menées en Afrique sub-saharienne sur le sujet sont essentiellement quantitatives [7, 9, 11, 12]. De plus, ces études ont concerné à la fois le milieu urbain et le milieu rural. Le milieu rural abrite la majorité de la population africaine et est associé à une faible prévalence contraceptive chez les femmes en union comparativement au milieu urbain [11] et mérite de ce fait d’être étudié plus spécifiquement. Aussi, les quelques études qualitatives déjà réalisées sur le sujet en Afrique ont analysé partiellement les déterminants de l’utilisation des contraceptifs modernes par les couples sans analyser les interrelations entre ces déterminants [13-15]. Enfin, peu de données existent sur le potentiel des politiques de gratuité de la PF sur les autres déterminants majeurs de l’adoption de la contraception moderne par les couples. L’objectif principal de notre étude était d’analyser les facteurs favorisant ou entravant l’utilisation d’un bon contraceptif gratuit par les femmes rurales.

## Méthodes

**Design de l’intervention du projet « analyse économique du comportement en santé de la reproduction au Burkina Faso » (BEARH-BF):** le BEARH-BF a été mis en œuvre dans trente (30) villages de la province du Houet, dans la région des Hauts-Bassins au Burkina Faso. Six (6) des treize (13) communes de cette province ont été tirées de façon aléatoire. Il s’agit de Faramana, Fo, Satiri, Lena, Karangasso-Sambla et Bobo Dioulasso. Dans chaque commune sélectionnée, cinq (5) villages ont été tirés de façon aléatoire. L’intervention a consisté à des entretiens avec les femmes éligibles sélectionnées suivis d’une distribution de bons contraceptifs gratuits sans counseling, ni diffusion de messages en faveur de la PF. Les critères d’éligibilité des femmes étaient les suivants: 1) être en couple, 2) vivre avec son mari ou son conjoint, 3) être âgée de 18 à 40 ans, 4) ne pas être enceinte, 5) ne pas être stérile, 6) ne pas avoir un problème de santé qui contre-indique l’utilisation d’un contraceptif moderne à l’étude et 7) donner son consentement éclairé pour participer à l’étude. La répartition des participantes selon le type de traitements et les échantillons correspondants sont présentés dans le Tableau 1. Les femmes bénéficiaires de la partie A (non utilisatrices) devaient utiliser elles-mêmes leur bon contrairement aux bénéficiaires de la partie B (utilisatrices) qui étaient invitées à transférer leur bon à un(e) ami(e) ou un(e) parent(e) non utilisateur(trice) du même village. Le bon donnait droit à un contraceptif (dispositif intra-utérin (DIU), implant, injectable ou pilule) au choix dans un centre de santé partenaire. Le délai de validité du bon était de trente (30) jours à compter de la date de sa remise. Six (6) mois après l’intervention, nous avons réalisé une enquête qualitative afin d’analyser les raisons de l’utilisation ou de la non-utilisation des bons. Cette enquête a concerné seulement les femmes de la partie A car l’équipe de recherche ne disposait pas de moyens de vérification pour les transferts de bons de la partie B.

**Tableau 1:** traitements et taille de l’échantillon

Personne qui reçoit le bon	Monogame	Polygame
**Partie A: couples où la femme n’utilise pas de contraceptif moderne**		
Femme en privé	300 (1)	300 (3)
Couple	300 (2)	300 (4)
**Partie B: couples où la femme utilise un contraceptif moderne**		
Femme en privé	400 (5)	-
Mari en privé	400 (6)	-

(1) Traitement 1; (2) Traitement 2; (3) Traitement 3; (4) Traitement 4; (5) Traitement 5; (6) Traitement 6

**Type d’étude:** il s’est agi d’une étude qualitative transversale avec un volet communautaire et un volet formation sanitaire.

**Populations d’étude:** la population d’étude pour l’enquête communautaire était constituée des couples non utilisateurs de contraceptifs modernes et qui ont reçu un bon. Celle de l’enquête dans les centres de santé était constituée par tous les agents de santé ayant participé à l’intervention dans les centres de santé partenaires.

**Echantillonnage et tailles des échantillons:** en recherche qualitative ce sont les critères de diversification et de saturation qui sont primordiaux pour déterminer la taille de l’échantillon [16]. La diversification intervient dans le choix de l’échantillon et la saturation dans la détermination de sa taille. Au moins quatre (4) participantes étaient sélectionnées de façon raisonnée par village en les diversifiant selon l’utilisation ou non du bon et le type d’union (monogame ou polygame). Quant aux entretiens au niveau des centres de santé, il était prévu d’enquêter un agent de santé par formation sanitaire soit 18 entretiens au total. Les critères d’éligibilité pour la sélection raisonnée des agents de santé étaient les suivants: 1) travailler dans un centre de santé partenaire au moment de l’intervention, 2) avoir assuré le service de PF au moment de l’intervention, et 3) avoir reçu au moins une femme avec un bon du projet BEARH-BF.

**Techniques et outils de collecte des données:** la collecte des données s’est déroulée du 18 décembre 2018 au 09 janvier 2019. Deux (2) focus groups (FG) ont été réalisés dans chaque commune dont un (01) FG avec les femmes qui ont utilisé leurs bons et un autre avec celles qui n’ont pas utilisé leurs bons. Douze (12) femmes étaient invitées pour chaque FG. Deux enquêtrices expérimentées dans la collecte de données qualitatives ont été formées pour la réalisation et la retranscription des entretiens individuels et des FG. Les entretiens individuels approfondis (EIA) ont été conduits auprès des agents de santé dans les centres de santé et les FG dans des endroits appropriés des communes. Un guide semi-structuré d’EIA et un autre de FG conçus pour atteindre les objectifs de l’étude ont été utilisés lors des entretiens. Les EIA et les discussions de groupes ont fait l’objet de prises de notes, d’enregistrements à l’aide de dictaphone numérique avant d’être intégralement retranscrits.

**Analyse des données:** il s’est agi d’une analyse thématique manuelle de contenu par simple catégorisation. Nous avons utilisé Excel et STATA version 13.0 pour le traitement et l’analyse des informations sociodémographiques des participantes aux FG.

**Considérations éthiques:** le protocole de recherche du projet BEARH-BF a été approuvé par le comité d’éthique pour la recherche en santé du Burkina Faso.

## Résultats

### Caractéristiques sociodémographiques des populations étudiées

**Participantes aux focus groups:** au total, 12 FG ont été réalisés avec au moins 10 participantes par FG. Le Tableau 2 montre les caractéristiques sociodémographiques des participantes aux FG. L’âge médian des femmes était de 29 ans avec un intervalle interquartile (IQR) de 8 ans. Le nombre médian des enfants par femme était de 3,37 ans avec un IQR de 2 ans. La plupart des participantes était des femmes sans niveau d’éducation (76,23%) et de religion musulmane (63,93%). Plus des deux-tiers des femmes avaient reçu le bon à l’insu de leurs maris soit 68,85%.

**Tableau 2:** caractéristiques sociodémographiques des participantes aux focus groups

Variable	Médiane ou pourcentage	IQR	Minimum	Maximum
Age (ans)	29	8	18	40
Nombre d’enfants vivants par femme	3,37	2	0	10
**Niveau d’étude**				
Aucun	76,23	-	-	-
Primaire	16,39	-	-	-
Secondaire	7,38	-	-	-
**Religion**				
Catholique	19,67	-	-	-
Musulmane	63,93	-	-	-
Protestante	9,84	-	-	-
Religion traditionnelle	5,74	-	-	-
Sans religion	0,82	-	-	-
**Type d'union**				
Monogame	55,83	-	-	-
Polygame	44,17	-	-	-
**Mode de remise du bon**				
Femme	68,85	-	-	-
Couple	31,15	-	-	-

IQR: Intervalle interquartile

**Agents de santé:** seize (16) agents de santé ont été interviewés dont neuf (9) femmes et sept (7) hommes. Ils étaient composés de sept (7) accoucheuses auxiliaires, quatre (4) sages-femmes/maïeuticiens d’Etat, quatre (4) infirmiers et un (1) agent itinérant de santé. La plupart de ces agents de santé avait entre 1 et 3 ans d’ancienneté à leur poste actuel. La plupart des agents de santé interviewés était responsable du service de maternité de leur centre de santé.

**Facteurs favorisant l’utilisation des bons:** quatre (4) principales raisons, non exclusives mutuellement, soutenaient l’utilisation des bons par les femmes: 1) les croyances aux avantages de la PF; 2) la gratuité des contraceptifs; 3) l’approbation du mari; 4) l’obligation morale.

**Croyances aux avantages du planification familiale:** les croyances aux avantages de la PF pour elles-mêmes, leurs enfants, leur mari et pour la société entière ont motivé plusieurs femmes à utiliser leur bon. Les déclarations suivantes de participantes aux FG le montrent: *« C’est pour espacer les naissances. Si les enfants ne sont pas espacés, la mère persiste dans la souffrance. […]. Elle devient pauvre »* (P7FG5, 24 ans, 02 enfants, union monogame, bon remis au mari). P7FG5 désigne le code de la femme.

*« C’est pour éviter les petites maladies chez l’enfant. Aussi, c’est pour que nous-mêmes nous soyons en bonne santé. Si ton enfant ne marche pas et tu tombes enceinte, l’enfant va souffrir, son père va souffrir, toi-même tu vas souffrir […] »* (P6FG5, 32 ans, 3 enfants, union polygame, bon remis à la femme en privé).

Comme l’a souligné un agent de santé, quelques agents de santé étaient aussi du même avis: *« […]. Celles qui sont venues, c’est parce qu’elles ont su qu’il y a une importance dans la planification […] »* (AS6, accoucheuse auxiliaire, inférieur à 1 an d’ancienneté). AS6 représente le code de l’agent de santé.

**Gratuité des contraceptifs:** à l’image de ces deux participantes aux FG, certaines femmes ont utilisé le bon à cause de la suppression de la barrière financière:

*« Si j’ai utilisé le bon, c’est parce que je voulais faire une méthode, mais je n’avais pas encore eu l’argent. Quand on m’a remis le bon, je suis vite allée au centre de santé pour faire […] »* (P2FG11, 35 ans, 03 enfants, union polygame, bon remis à la femme en privé).

*« Je désirais faire une méthode mais je n’avais pas de moyen financier pour ça. Vous êtes venus avec la gratuité, ça m’a vraiment soulagé »* (P4FG11, 30 ans, 04 enfants, union monogame, bon remis à la femme en privé).

La majorité des agents de santé était du même avis comme en témoigne cette déclaration d’un infirmier: *« […] nous avons remarqué que c’est l’accès financier qui bloque les femmes. Donc, celles qui étaient dans le désir et qui étaient bloquées financièrement, pour elles ces bons-là étaient les bienvenus […] »*(AS9, infirmier, 1 an d’ancienneté).

**Approbation du mari:** plusieurs femmes ont déclaré avoir utilisé leur bon suite à l’approbation de leur mari comme l’indiquent les témoignages ci-dessous: 1) *« Quand on m’a remis le bon, mon mari était présent, il m’a dit de partir le lendemain au centre de santé faire une méthode pour espacer mes naissances […]. Moi-même je ne peux pas décider […] »* (P8FG11, 34 ans, 03 enfants, union monogame, bon remis au mari).

*« […] Le fait qu’il y a eu la communication en famille, le chef de ménage a accepté […]. Si on avait des occasions comme ça, on sait que ça va donner encore plus […] »* (AS16, sage-femme).

**Obligation morale:** quelques femmes ont déclaré avoir utilisé le bon par conscience personnelle sans y être forcées comme l’attestent les déclarations ci-dessous: 1) *«Comme ils m’ont remis le bon, si je n’utilise pas, cela n’est pas bien. C’est pour cette raison que j’ai utilisé le bon »* ( P2FG5, 19 ans, 0 enfant, union monogame, bon remis à la femme en privé).

2) *« C’est vous qui nous avez dit d’aller au centre de santé. Raison pour laquelle nous sommes allées […] »* (P5FG5, 38 ans, 2 enfants, union monogame, bon remis à la femme en privé).

**Facteurs entravant l’utilisation des bons:** de l’analyse des contenus des FG et des EIA, les cinq (5) facteurs suivants ont été identifiés: 1) le désir de tomber enceinte; 2) l’opposition du mari; 3) la réticence des femmes; 4) la méconnaissance des contraceptifs modernes; 5) les facteurs liés à l’intervention. Les résultats détaillés de chacun de ces facteurs sont présentés ci-dessous.

**Désir de tomber enceinte:** plusieurs femmes ont déclaré n’avoir pas utilisé leurs bons parce qu’elles désiraient tomber de nouveau enceinte. C’est le cas de ces deux (2) femmes: 1)*« Mon dernier enfant est grand. Donc je désire un autre enfant. Raison pour laquelle je ne suis pas allée au centre de santé avec le bon »* (P11FG2, 32 ans, 02 enfants, union polygame, bon remis à la femme en privé).

2) *« Quand on me remettait le bon, mon enfant avait déjà 04 ans et je voulais un autre enfant. C’est pour cela que je n’ai pas utilisé le bon »* (P5FG3, 27 ans, 04 enfants, union polygame, bon remis à la femme en privé).

**Opposition du mari:** comme l’illustrent les récits ci-dessous, plusieurs femmes n’ont pas utilisé le bon à cause du refus de leurs maris: 1) *« Mon mari n’a pas accepté que j’aille adopter une méthode contraceptive »* ( P12FG2, 21 ans, 01 enfant, union polygame, bon remis à la femme en privé ).

2) *« J’ai reçu le bon, mais je ne pouvais pas l’utiliser sans l’accord de mon mari. Je suis restée dans la négociation jusqu’à ce que la date limite arrive. Et je n’ai pas eu son accord. […] »* (P2FG8, 36 ans, 03 enfants, union polygame, bon remis au mari).

La plupart des agents de santé interviewés étaient aussi du même avis comme en témoigne la déclaration ci-dessous:

3) *« […] Souvent, les conjoints ne sont pas pour la PF. Ce qui fait que certaines femmes ne viennent pas, surtout les musulmans, ils refusent. Ils disent que dans le coran c’est interdit »* (AS13, maïeuticien d’Etat, 03 ans d’ancienneté). Diverses raisons principalement d’ordre religieux et ethnique ont été évoquées par les participants pour justifier le refus des maris.

**Réticence des femmes:** les idées reçues et les effets secondaires sont les principales raisons qui ont été évoquées par les participants pour expliquer la réticence des femmes vis-à-vis des contraceptifs modernes. Les déclarations ci-dessous de certains participants font échos de cela: 1) *« Moi j’ai déjà utilisé une méthode et ça m’a beaucoup fatigué. J’ai fait un accident et j’ai souvent mal au dos. Avec la méthode que je faisais, mes menstrues coulaient trop et cela aggravait mes maux de dos. […]. J’ai dû arrêter la méthode suite à cela […] »* (P6FG3, 22 ans, 01 enfant, union polygame, bon remis à la femme en privé).

3)*« […] Elles avaient peur des effets secondaires du produit. Par exemple, on leur dit: « si tu mets, après ça disparait dans ton corps ». Il y a d’autres qui disent: « si tu mets, les règles viennent et ça ne se coupe pas […] »* (AS11, infirmier, 2 ans d’ancienneté).

**Méconnaissance des contraceptifs modernes:** le manque d’informations des femmes sur les contraceptifs modernes est aussi une des raisons de la non utilisation des bons comme l’atteste la déclaration ci-après : 1)*« […] Il y a beaucoup de femmes qui viennent (pour d’autres services de santé que la PF) et qui sont à leur 4^e^ou 5^e^enfant et qui n’ont jamais fait de PF […]. Ça veut dire que quelque part elles n’ont pas tellement de connaissances sur la PF […]. Je pense que réellement elles n’ont pas été sensibilisées sur ça »* (AS11, infirmier, 2 ans d’ancienneté).

**Facteurs liés à l’intervention:** la plupart des agents de santé interviewés ont évoqué des facteurs intrinsèques à l’intervention qui expliqueraient la non-utilisation des bons. Il s’agit principalement de (du):1) l’incompréhension des femmes concernant l’usage des bons; 2) la faible, voire la non implication des hommes et de certains acteurs locaux dans l’intervention; 3) la période peu favorable de l’intervention; 4)le délai de validité du bon jugé très court; 5) l’imposition d’un seul centre de santé à la femme pour l’utilisation de son bon.

Les opinions d’agents de santé ci-dessous illustrent certains de ces facteurs: 1) *« […] Parce qu’il y a d’autres (femmes) qui sont venues dire que le bon est là et qu’elles ne savent pas ce qu’il faut faire avec […] »* (AS15, sage-femme, 1 an d’ancienneté).

2) *« […] Selon leurs dires (des femmes) c’était le temps parce que ça a coïncidé avec les travaux champêtres, elles n’avaient même pas un petit temps pour venir ici […] »* (AS5, accoucheuse auxiliaire, 02 ans d’ancienneté).

## Discussion

Cette étude avait pour principal objectif d’analyser les facteurs favorisant ou entravant l’utilisation d’un bon contraceptif gratuit par les couples en milieu rural au Burkina Faso. Les données issues de notre enquête indiquent que le coût des contraceptifs même « dérisoire » demeure un obstacle financier à l’adoption de la contraception moderne par certaines femmes en milieu rural. Aussi, la seule suppression de la barrière financière n’est pas suffisante pour agir sur les autres obstacles majeurs connus à l’adoption de la contraception moderne par les couples en milieu rural.

**Le coût des contraceptifs modernes, cause de besoins non satisfaits en planification familiale:** au Burkina Faso, avec la subvention, les femmes dépensent 250 à 500 FCFA pour un DIU, un implant ou un injectable soit 0,72% à 1,44% du salaire minimum interprofessionnel garanti (SMIG) et 50 FCFA pour une plaquette de pilules soit 0,14% du SMIG sans compter les coûts du déplacement et souvent des consommables médicaux pour certains contraceptifs. Le SMIG étant de 34 664 FCFA au Burkina Faso [17]. Même ces coûts à priori «dérisoires», constituent la principale raison de la non adoption de la contraception moderne par certaines femmes ayant participé à notre étude dans un pays où près de la moitié de la population rurale vivait en dessous du seuil de pauvreté en 2014 [18]. En 2017, Machiyama *et al*. au Kenya et au Bangladesh avaient aussi trouvé que le coût des contraceptifs était une des raisons des besoins non satisfaits en PF [6]. Toutefois, la gratuité de la PF à elle seule n’est pas suffisante pour lever les autres obstacles majeurs à l’utilisation des contraceptifs modernes par les femmes rurales en union. D’acteurs facteurs non des moindres sont à considérer.

**Les autres déterminants majeurs de l’utilisation des bons gratuits:** l’accord des maris a été un facteur important de l’utilisation du bon par leurs femmes. Ce résultat est similaire à ceux d’autres études antérieures menées en Afrique et qui avaient trouvé que l’approbation des maris est un déterminant à l’adoption de la contraception moderne par leurs femmes [7, 10, 13, 19]. Cette situation s’explique par le fort pouvoir de décision des hommes et leurs préférences de fertilité plus élevées comparativement aux femmes surtout en milieu rural. Mosha *et al*. en 2013 en Tanzanie, Corroon *et al*. en 2014 au Nigeria et Farmer *et al*. en 2015 au Rwanda ont montré la forte influence et la domination des hommes dans la prise de décisions en matière de fécondité [15, 19, 20]. Au Burkina Faso, les hommes sont globalement plus natalistes que les femmes car le nombre moyen idéal d’enfants des hommes en union est plus élevé que celui des femmes en union (7,4 enfants contre 5,8 enfants) [21]. En plus des préférences de fertilité très élevée des hommes, les influences religieuses, ethniques et coutumières ont été évoquées par les participants à l’étude pour justifier le refus des hommes.

Les bons ont été remis aux femmes sans counseling, ni sensibilisation préalable sur la PF et plusieurs femmes ont déclaré avoir utilisé leurs bons pour les nombreux avantages de la PF. Il s’agit donc de femmes déjà sensibilisées sur la PF. En 2015, Eshete en Ethiopie avait aussi trouvé que la connaissance sur les méthodes contraceptives était un des facteurs significatifs associés à leur utilisation par les femmes en union [11]. Lorsque la femme est sensibilisée sur la PF, cela accroît sa propension à adopter la contraception moderne. Elle connait les avantages de la PF, elle croit moins aux fausses idées reçues et elle est plus rassurée concernant les éventuels effets secondaires des contraceptifs modernes. A l’image d’autres études antérieures menées en Afrique [7, 10, 11], le désir de la femme de tomber enceinte est un déterminant de la non utilisation du bon. Les femmes ont des préférences de fertilité toujours très élevées en Afrique sub-saharienne en général et au Burkina Faso en particulier. Au Burkina Faso, le nombre moyen d’enfants en 2010 était de 5,8 enfants chez les femmes en union âgées de 15 à 49 ans. Parmi ces femmes, 71% désiraient plus d’enfants [21]. Aussi, les femmes burkinabè en milieu rural ont un niveau de fécondité nettement plus élevé que celles du milieu urbain (6,7 enfants contre 3,9 enfants par femme) [21].

La réticence des femmes vis-à-vis des produits contraceptifs était un important facteur de la non-utilisation des bons. Cette réticence des femmes se justifiait principalement par la peur des effets secondaires des contraceptifs modernes comme l’ont montré d’autres auteurs avant nous [10, 13, 14]. En plus des effets secondaires des contraceptifs modernes, notre étude a révélé l’impact des fausses idées sur la réticence des femmes quant à l’adoption de la contraception moderne. Enfin, des facteurs inhérents à la conception et la mise en œuvre de l’intervention ont justifié la non-utilisation du bon par certaines femmes. Ce résultat est nouveau comparativement à ceux des études antérieures sur le sujet. L’évaluation du processus, depuis la conception jusqu’à la mise en œuvre, de toute intervention de santé publique est le seul moyen d’identifier ce type de facteurs afin d’améliorer la mise en œuvre des interventions en cours ou futures.

Notre étude présente les limites suivantes: 1) il est possible qu’il ait eu une contamination entre notre intervention et la semaine nationale de la PF qui venait de prendre fin trois (03) jours plus tôt; 2) les raisons des oppositions des maris n’ont pas été suffisamment investiguées dans le cadre de notre étude; 3) notre étude a été menée dans une province du Burkina Faso. Elle n’est donc pas généralisable à tout le pays qui compte au total 45 provinces.

## Conclusion

Notre étude a mis en évidence le fait que la suppression de la barrière financière n’est pas suffisante pour lever les autres barrières majeures à l’utilisation des services de PF par les femmes en union. Par exemple, les facteurs contextuels et interventionnels demeurent déterminants dans l’utilisation des contraceptifs modernes par les femmes en union résidant en milieu rural même en situation de gratuité des services de PF. Ainsi, pour une augmentation significative et durable de la prévalence contraceptive au Burkina Faso, la stratégie nationale de gratuité de la PF devra être soutenue par des stratégies éprouvées d’implication des hommes à la PF en complément de stratégies d’autonomisation et d’instruction des femmes et des jeunes filles afin de baisser leurs préférences de fertilité toujours élevées.

### Etat des connaissances actuelles sur le sujet

- Le taux des besoins non satisfaits en PF reste encore élevé dans les pays d’Afrique sub-saharienne et au Burkina Faso;
- Le refus des maris et les préférences de fertilité encore élevées des femmes en union surtout en milieu rural en Afrique sub-saharienne constituent les principaux facteurs de la faible prévalence contraceptive dans ces pays.

### Contribution de notre étude à la connaissance

- Notre étude renseigne sur le potentiel de la nouvelle politique de gratuité de la planification familiale avant même son avènement au Burkina Faso; elle montre que la gratuité des soins et des produits contraceptifs seule n’a pas un effet dominant sur les autres déterminants majeurs de l’adoption de la contraception moderne par les couples en milieu rural;
- Par conséquent, notre étude suggère que la promotion de la contraception moderne au profit des couples surtout en milieu rural requiert une approche holistique associant à la fois a) la gratuité des soins et des produits contraceptifs, b) le développement de stratégies boostant l’implication des hommes dans la PF et c) le développement de stratégies faisant baisser les préférences de fertilité toujours élevées des femmes et des hommes en union;
- Enfin, notre étude renseigne sur la nécessité d’évaluer les processus des interventions de planification familiale pour identifier, entre autres, les insuffisances inhérentes à leur processus d’implémentation aux fins de les corriger.

## Remerciements

L’équipe de recherche remercie très sincèrement les autorités administratives, sanitaires, coutumières et religieuses des communes et villages sites du projet, les participants à l’étude, le personnel de terrain, les personnes ressources en santé de la reproduction des Hauts-Bassins, le Conseil de Recherche Économique et Social (ESRC) et le Département pour le Développement International (DFID) du Royaume Uni (ESRC-DFID grant ES/N014618/1) pour le financement du projet BEARH-BF.
